# The call for an “educational footprint” in conceptualizing mental health and psychosocial support: the case of Norwegian kindergarten teachers

**DOI:** 10.3389/fpsyg.2024.1339332

**Published:** 2024-05-30

**Authors:** Ingrid R. Christensen

**Affiliations:** University of South-Eastern Norway (USN), Notodden, Norway

**Keywords:** mental health and psychosocial support, kindergarten, teachers, conceptualization, qualitative study mental health and psychosocial support, qualitative study

## Abstract

This article presents an in-depth qualitative case study asking, “How do Norwegian kindergarten teachers conceptualize and negotiate the field of psychosocial support?” This article thoroughly explores how Norwegian kindergarten teachers perceive of the field Mental health and psychosocial support (MHPSS). Recognizing the global imperative to advance mental health and psychosocial support for children, the study highlights the crucial need for interprofessional grounded concepts and logics in developing MHPSS interventions. The study employs a grounded theory approach to actively develop concepts in the MHPSS field in Norway. The six selected Norwegian expert teachers can make up a so-called “unique case”, representing a high-income country with a robust welfare system and child-centered pedagogy. Yet, they reveal challenges in embracing the concept of psychosocial support: The analysis shows that the teachers seem to neglect and even reject the concept of psychosocial support, while also expressing inferiority and lack of agency towards other professions. However, the teachers also express the desire for more knowledge, and with proudness they energetically engage in fostering children’s agency and functioning by their everyday descriptions of «meeting with the child». The study calls for the kindergarten teachers’ increased efforts to actively participate in MHPSS interventions, advocating for the further development of an «educational footprint» that can empower interprofessional work.

## 1 Introduction

This article presents a qualitative case study that investigates how kindergarten teachers in Norway conceptualize and negotiate the field of mental health and psychosocial support (MHPSS). The Norwegian context is particularly interesting due to its status as a high-income country and a stable welfare system. The kindergarten has in general highly qualified teachers and a strong program for child-centered pedagogy. This paper therefore asks: *How do Norwegian kindergarten teachers conceptualize and negotiate the field of psychosocial support*?

The global need for increased attention to the field of mental health and psychosocial support (MHPSS) for children is globally acknowledged. The rising instabilities caused by political conflicts, a shared economic crisis, a pandemic, and environmental changes have amplified concerns about the mental health and well-being of children worldwide (Collishaw and Sellers, 2020; Hossain et al., 2022; World Health Organization, 2022b). The field of mental health and psychosocial support comprise measures directed toward “any type of local or outside support that aims to protect or promote psychosocial well-being and/or prevent or treat mental health condition” (UNHCR, 2022).

During the last decade, there is an increased awareness of children’s mental health in schools and kindergartens (Holen and Waagene, 2014; Tjomsland et al., 2021; World Health Organization, 2021, 2023). Education, and especially kindergarten, is an arena with tremendous potential for providing psychosocial support with available professional competence in everyday relations (Soneson et al., 2020; World Health Organization, 2023) While a teacher’s role does not officially include being a mental health practitioner, education should provide learning and well-being for all students (Andrews et al., 2014).

As a consequence, MHPSS is implemented in the formal curricula of education in many countries (Cefai et al., 2021). The kindergarten is considered effective for “early prevention” measures (Holmes et al., 2015). In Norway, MHPSS has been part of the national curricula in schools as well as the kindergarten framework plans in education since 2017 under the label “public health and life skills” (Ministry of Education and Research, 2017), and is supplemented by programs and teaching materials for use in public and private kindergartens. Many of these Norwegian measures often take a cognitive-psychological approach, describing emotional regulation as the teachers’ most important task (Nordanger and Braarud, 2014, Bru et al., 2016, p. 22).

However, the implementation of the field of MHPSS in education meet several common challenges across many countries. International and Norwegian evaluations of interventions show limited long-term effects of interventions and programs (Gjerustad et al., 2019; Maynard et al., 2019; Finning et al., 2020). Some explanations for the shortcomings are explained as matters of implementation methods, such as developing feasible measures (Soneson et al., 2020). Some pinpoint the need for a more standardized conceptual basis in the programs (Bangpan et al., 2016; Augustinavicius et al., 2018). Some shortcomings of the MHPSS can be explained as a lack of consideration of the gaps between knowledge, services, and adjustments to actual local needs (Fatori and Polanczyk, 2020; Soneson et al., 2020). Furthermore, some emphasize the lack of interprofessional collaboration across community sectors such as health, social services, and education as a reason for possible failures of effective interventions (Lund et al., 2013; Sashidharan et al., 2016).

In addition, one main criticism is that the rationale and many of the concepts in MHPSS interventions in education by and large are developed by the health sector. The MHPSS field is dominated by psychological or psychiatric definitions and measures, and with none or few contributions from the educational sector. Some studies of MHPSS in education show that teachers tend to question their mandate and role in dealing with mental health issues, and in addition, they express that they do not have the formal competence to do so (Stubø, 2018; Ní Chorcora and Swords, 2021). Furthermore, educational programs are not providing enough preparation for teachers, and teachers express that they do not have enough knowledge to position themselves toward materials produced by interest groups (Andrews et al., 2014; Shelemy et al., 2019). The teachers’ own contribution to interventions seems to be rare, if not absent (Maynard et al., 2019).

Thus, there is a need for developments that can balance systematized and standardized measures of MHPSS *and* local, interprofessional interventions (Jordans et al., 2016; Haroz et al., 2020; Kamali et al., 2020). There is also a need for bottom-up contributions for developing a particular “educational footprint” that adds to the field of MHPSS by investigating the logic and concepts of MHPSS among kindergarten teachers. Against this background, this study explores how kindergarten teachers themselves conceptualize and negotiate the field of psychosocial support.

The present study is a development of a previous international comparative study of psychosocial support described by kindergarten teachers in Palestine and Norway. The comparison showed that although Palestinian kindergarten teachers were more well-acquainted with the MHPSS terminology, the two countries shared many common challenges in providing psychosocial support in kindergarten. The study describe a need for further national studies to develop a more distinct “educational footprint” in the field of MHPSS (El-Khodary et al., 2021). The aim of this present paper is to bring in-depth insights into teachers’ professional logic and conceptualization of psychosocial support, calling for an “educational footprint” in interprofessional work in MHPSS interventions.

In the following sections, I will describe the case of kindergarten and the field of MHPSS in Norway, and some key concepts and methodological considerations of the study before an in-depth analysis of the kindergarten teachers’ conceptualizations and negotiations of the field of psychosocial support.

## 2 The case of Norway: the relevance of exploring the field of psychosocial support in Norwegian kindergartens

The question about how kindergarten teachers describe psychosocial support toward traumatized children is a key concern in Norway countries, along with the challenges of mental health, early intervention, and the particular role of kindergarten. Norway shares the complex global challenge of an increasing number of young people seeking help for mental health difficulties (Nordic Council of Ministers, 2017). Even though Norway represents a stable high-income country and a well-founded general welfare, psychosocial difficulties are considered among the largest challenges for public health (Department of Health and Care, 2019). In Norwegian kindergartens, 4–7% of the children require mental health services (Størksen et al., 2018).

On this background, the study from Norway might represent a contribution to the field of MHPSS as a “best case scenario” or a “unique case” (Yin, 2009, pp. 46–47). Norway has a generally stable national infrastructure, a strong public kindergarten, an overall high formal competence among the teachers, and also good material conditions. Enrolment into ECEC before formal schooling is 97% of children between 3 and 5 years old in Norway (Norwegian Directorate for Education and Training, 2023). All children in Norway have an individual right to center care from age one, and about 90% of the children attend a kindergarten on a full-day basis, the majority from the age of 1-year-old (Norwegian Directorate of Education, 2019). As a basis, children are offered kindergarten with publicly regulated rates, being subsidized by the government, based on the equal right to education and early intervention (Einarsdottir, 2011; Haug, 2013). Initiatives such as discount schemes and free core time aim to boost kindergarten enrollment. Both regionally and decentralized strategies and supervision for professional development actively support local quality of the kindergarten (Meld.St.6, 2019-2020). Over 40% of Norwegian kindergarten staff hold a 3-year bachelor’s degree in early childhood education and care, while other staff typically complete shorter vocational courses or gain experience.

Both public and private kindergartens in Norway are regulated by the Kindergarten Act (Ministry of Education and Research, 2017), and their purpose and content adhere to the national curriculum known as The Framework Plan for the Content and Tasks (Norwegian Directorate of Education and Training, 2017). The Framework Plan for Kindergartens clearly defines the roles and responsibilities of kindergartens, as well as underlining the rights of children and parents. The Framework plan suggest a core staff ratio-to children, requiring one adult for every three children under 3 years old and one adult for every six children over 3 years old.

Norwegian kindergartens prioritize free play as an inherent aspect of childhood, as an activity and end goal in itself, adopting a holistic approach to children’s development in close collaboration with the parents. The ideas presented in the data material can be interpreted in the light of general Norwegian pedagogical ideals of child-centered pedagogy, representing a particular childhood ideal of independence and self-governed development prior to academic learning. Children should be viewed as “beings” and not only “becomings,” and the teacher should value a child’s own agency and take time for their initiatives (Bae, 2007).

Since 2017, MHPSS has had the label “public health and life skills” in the framework plan for kindergarten (Ministry of Education and Research, 2017), In Norway MHPSS programs have had increased attention after the covid pandemic (Nøkleby et al., 2021), and is supposed to support the formal curricula in education. For instance, in some parts of Norway there has been a particular political priority to develop “Trauma sensitive kindergartens.”

However, there are sharp controversies within educational context about the framing of MHPSS. On the one hand, there is a political priority of introducing the measures of MHPSS through the Framework plan, followed by financial support for several MHPSS interventions in kindergartens. On the other hand, representatives of education raise critical questions about the relevancy of MHPSS interventions in kindergartens, especially if they promote coming and mastery of life as a “competence,” as if life itself can be measured, tested and failed (Helskog, 2019; Madsen, 2020). They argue toward education as a therapeutic and clinical arena and seem to oppose *any* kind of standardization or programs, based on the conviction that MHPSS in terms of coping and mastery of life puts an even higher pressure on children today. On this basis, there should be good conditions for developing the field of MHPSS in Norwegian kindergartens, yet the discursive academic landscape shows that this can be contested.

## 3 The concept of mental health and psychosocial support

This study explores kindergarten teachers’ own conceptualizations and negotiations of psychosocial support, aiming at contributing to an “educational footprint” in interprofessional work in the field of MHPSS. The Inter-Agency Standing Committee (IASC) describes the field of psychosocial support as the support provided by a community and holds that “the composite term mental health and psychosocial support is to […] describe any type of local or outside support that aims to protect or promote psychosocial well-being and/or prevent or treat mental disorder” (Inter Agency Standing Committee, 2022). This description can be supplemented with Woodward’s (2015) definition, holding that psychosocial support should include “the combined influence that psychological factors and the surrounding social environment have on children’s physical and mental wellbeing and their ability to function.”

The descriptions of psychosocial support of IASC and Woodward are wide, flexible and comprise psychosocial support as a field of practices and an open space for the teachers’ own conceptualizations. At the same time, the description of psychosocial support also sets a focus on the interactional quality of MHPSS and specific social patterns and transactions between the child and their caregivers. Additionally, they underline that MHPSS should rely on interprofessional work and the collective cooperation rather than being a domain of for instance the health sector. The interplay among different societal actors significantly impacts a child’s physical and mental well-being, as well as their ability to function in society (Woodward, 2015). Individuals, families, kindergartens, the environment, municipal administration, societal structures, and the political situation collectively influence a child’s development (Bronfenbrenner and Morris, 1998; Sabol and Pianta, 2012).

Although this study adheres to the larger field of MHPSS, the main analytical unit of this study is «psychosocial support», as many Norwegian teachers themselves seemingly contest their role within the mental health field (Stubø, 2018). The concepts of mental health could have created distance, or in worst case, be seen as irrelevant for the informants. Another reason for this is that present study does not cover the field of MHPSS for instance clinical assessment, treatment, or describe clinical trauma as such. By using the term “conceptualize,” I will turn the attention to the combination of the teachers’ own terminology and the contexts making a meaningful conceptualization of psychosocial support. By “negotiation,” I open up for the inclusion of interpretations, tensions, priorities, and values that kindergarten teachers express. Exploring the conceptualizations and negotiations of kindergarten teachers may catalyze further development of an educational contribution and “footprint” in interprofessional work within the field of MHPSS.

### 3.1 Methods of the study

This paper offers a new and in-depth analysis of the Norwegian segment of data, being part of a previous comparative study on kindergarten teachers’ perspectives on psychosocial support in Palestine and Norway (El-Khodary et al., 2021). The two studies shared certain features early in the research process, serving as a mutually informative reference group during various research phases. The prior study (El-Khodary et al., 2021) provided brief comparisons across two distinct national contexts, while this present study represents new analyses, research questions and units of analysis, calling for in-depth national studies of the data material.

The study holds a so-called “one-case design.” A case study can be described as a flexible and comprehensive methodology with well-established procedures (Creswell, 2009). A case study yields an opportunity for in-depth exploration of the phenomenon under study” (Mohammed et al., 2015, p. 100). In this article, we conduct a study of *one* phenomeon, namely the term psychosocial support, as it conceptualized by Norwegian kindergarten teachers. By treating it as a case, we can have knowledge about how the concept of psychosocial support appears in a specific period of time (2018), in time and context, and according to certain knowledge domains, kindergarten teachers. Studies like these cases can offer essential insights for the academic leadership of kindergartens and serve as a foundation for developing other research designs for larger samples.

As for sampling, this present study comprises a strategic sampled case of six well-educated kindergarten teachers with specialized experience in providing professional support for trauma-exposed children (Table 1).

**Table 1:** Description of the sample in the study.

	Education	Experience	In-service training in psychosocial support and/or regular supervision from public educational psychological services
Informant 1	2 Bachelor degrees, Kindergarten teacher and social work	App 10 years in kindergarten	Yes
Informant 2	MA in inclusive education and educational psychology	4 years in kindergarten	Yes
Informant 3	BA kindergarten teacher degree	5 years in kindergarten	Yes
Informant 4	2 BA degrees: BA child care and BA education.	<1 year in kindergarten	Yes
Informant 5	BA kindergarten teacher degree	22 years in kindergarten	Yes
Informant 6	No higher education	42 years in kindergarten	Yes

All the kindergarten teachers work in kindergartens in a city in the southeastern part of Norway with a mixed socio-economic environment, including a significant number of immigrants and refugees. This constitutes what can be labeled an “expert sample,” particularly in terms of higher education and in-service training.

Most of the teachers have comprehensive higher education. One holds a bachelor’s degree in early childhood, another is a kindergarten teacher with a bachelor’s in child welfare, and a third has a Master’s in Inclusive Education. Some of the teachers possess two higher degrees. Experience in kindergarten varies from a few months to 42 years.

In the evaluation the combination of the sample toward the responses, I reflected about whether education level, age, or having children themselves educational program, and years of experience are significant for the quality or the depth of the responses. Within the scope of this research design, it is not possible or relevant to draw conclusions on the background of singular factors of the sample.

Another factor I considered was whether employment in public or private kindergartens had an impact on the responses, which, in this context, was negative. Four teachers were from a public kindergarten, and the other two were from a private kindergarten. In Norway, the public kindergarten sector includes both public and private kindergartens, all subject to close management and adhering to the same quality systems, psychological supervision systems, and a standardized teacher/child ratio in both public and private kindergartens.

Therefore, on an overall level, the responses are developed as a one-case design, meaning that the responses are analyzed by their content, and not by person. The qualitative design still pays close attention to the details and logics of the informants’ responses, and tensions and contradictions in the data materiel. The aim of this study is to explore the qualitative conceptualizations and logics in a selected group of kindergarten teachers who are evaluated as highly qualified for describing the concept of psychosocial support.

Data were collected from September to October 2020 and selected through an open call and direct contact with kindergartens. In addition, many of the teachers work in multicultural areas, and with immigrants and refugees.

The interviews in this study were semi-structured. The interview protocol was not developed from pre-defined specified clinical indicators; instead, it poses open-ended interview questions regarding the teachers’ concepts and conditions for providing psychosocial support. A first question to the informants, all the informants was how they would define the term “psychosocial support.” It was followed up by description of how the teachers provide psychosocial support, as well as the children’s needs for psychosocial support, and then adjusted to the individual responses from the kindergarten teachers.

The interviews were conducted either face-to-face or on Zoom (2 face-to-face and 4 on Zoom). Data were gathered by a native-language-speaking researcher using a qualitative semi-structured interview guide. The semi-structured interview was dynamic, primarily structured around the question of how kindergarten teachers would describe the concept of psychosocial support, but also following the informant’s lead in connecting terms and engagement. All the interviews were transcribed and then translated into English, with the process overseen by a second native speaker. The respondents were given fictional names. From anonymity reasons, these are not the same as “Informant 1, 2, etc.

This present paper employs a grounded-theory analytical strategy (Glaser and Strauss, 1967), providing a “bottom-up” concept and offering “thick descriptions” (Geertz, 1983). The grounded theory approach was applied in two steps: During the first step, I conducted several thorough readings of the data material and outlined a few central keywords that appeared to be significant for all the kindergarten teachers, utilizing the strategy of so-called open coding (Strauss and Corbin, 1990, p. 61). These categories were not predefined but stemmed from the data material. As an example, the responses to the question “how do you define psychosocial support” were organized below each other, with comments provided for each, specifying the dimension to which they referred. As a result, the findings are presented as a one-case scenario denoting the common key tendencies among the kindergarten teachers. During the second step, I systematized paragraphs connected to the keywords and read them in their contexts, seeking to explore the format of the data material, their speech acts through more elaborate descriptions and engagements (so-called “axial coding,” Strauss and Corbin 1990, p. 123).

For instance, some teachers did not find all the questions relevant, including the term “psychosocial support,” which is crucial to describe in the analysis. On one hand, one could consider this a “zero finding.” On the other hand, it is important to conduct systematic reflections on how and why the teachers respond the way they do. Thus, the study seeks to identify unspoken presuppositions, implications, negotiations (Abrams and Harpham, 2005), and even subversions of professional logics (Freidson, 2001).

Considering the trustworthiness of qualitative research (Peels and Bouter, 2023), the current study involving Norwegian kindergarten teachers has been discussed within an international research group at various stages, including planning and discussing the specific context of MHPSS in Norwegian kindergartens. We discussed the interview guide connected to the Norwegian context and the selected informants and conducted initial analyses together. This collaborative effort resulted in the first paper, shedding light on the need for a new and in-depth analysis of the rich data from the Norwegian sample.

The study has adhered to the guidelines for data collection, information, and handling of data, and has been clarified according to the requirements of Norwegian Agency for Shared Services in Education and Research (sikt.no/en). The participants received invitations in written form with information about the study, of anonymization procedures and the rights to withdraw at any time.

As for the validity issues, this study will provide analytical exploration rather than statistical generalization (Flyvbjerg, 2001). The findings develop generalizations of topics and themes used to generate key notions and clarify key conditions for the further development the field of MHPSS in kindergartens in Norway and in an international context.

## 4 Analytical explorations: kindergarten teachers negotiating the concept of psychosocial support

This section introduces some key analyses and results of how Norwegian kindergarten teachers conceptualize psychosocial support, aiming at bringing in-depth insights into teachers’ professional logic and conceptualization of psychosocial support, calling for an educational contribution and “footprint” in interprofessional work in MHPSS interventions.

A dual analytical lens will be employed, showing the emphasized content of psychosocial support as well as showing *how* kindergarten teachers address the concept of psychosocial support. The analysis presents three main topics from the interviews with the kindergarten teachers: Kindergarten teachers’ rejection of the concept of psychosocial support, the reconceptualization and new components of psychosocial support, and the professional identity and positioning toward the field of psychosocial support.

### 4.1 Psychosocial support rejected by kindergarten teachers

An overall finding in this study, is that the concept of psychosocial support is weakly established. When the kindergarten teachers respond to the question about how they would describe the term “psychosocial support,” there is a strikingly similar response:

“It’s not a word we use in kindergarten, psychosocial support? Is that what you said? But we perform psychosocial support, as I understand the concept. In the form of supporting children’s mental health and interaction with others, self-regulation, taking the other's perspective … coping with feelings, considering the feelings of others, and expressing feelings. […] “I work mostly with children where the PPT [psychological-pedagogical services] has used the term” attachment difficulties or disorder”. Here we see a lot of quite strong feelings, an overwhelming expression of one or the other feeling. Preoccupied with feelings, one is not just a bit sad, but very sad …” (Kari)

“For me … well I am not used to using that concept. But building good relationships, supporting children in all situations, is not a such precise language, but is the same meaning”. (Brit)

“I have read your definition (laughs), in kindergarten, it is about meeting children’s emotional needs, that's what I think of first. To be available for children and create a safe space for the children. So that they dare to show their feelings and that they will meet adults that can cope with their feelings. No matter what one has experienced” (Anna)

“It is quite a wide concept, but essentially it’s about meeting the child where they are, on many levels”. (Ida)

In the first lines of the quotes, the kindergarten teachers seem to reject the term psychosocial support, by “it’s not a word we use …,” “I have read your definition (laughs) ….,” “I am not used to that concept …” “It is quite a wide concept …” These kindergarten teachers emphasize that the term psychosocial support is *not* a concept they immediately would identify as their own; that they are not used to it or criticizing it as a too wide concept. They underline the distance to it by repeating the definition given by the interviewer, laughing a bit, as if it is not precise or strange to their professional enterprise. They seem to comment with particular confidence, as if questioning, othering, and maybe even ridiculing the term psychosocial support does not apply to what the kindergarten teachers do. At first glance, psychosocial support as a concept does not hit the target for what these teachers think they do.

### 4.2 The “But” introduced: psychosocial reconceptualized

However, the quoted kindergarten teachers offer many alternatives to the term psychosocial support. Some of them do so by saying “but,” introducing a negotiation of the concept, and demonstrating a certain demarcation of a contrasting position. One could say that the kindergarten teachers now negotiate it and explain what they do, providing nuances and maybe even replacing psychosocial support as a concept.

After this introduction, the kindergarten teachers establish some components of the concept of psychosocial support: Kari enlists some concepts above that psychosocial support is to support children’s mental health and interaction with others, self-regulation, taking the other’s perspective … coping with feelings. Kari also mentions “attachment difficulties or disorder” - underlines that it is a term used by others, the public-school psychologist service (called “PPT”). Kari somehow sees the need for replacing the term with everyday language by saying “one is not just a bit sad, but very sad ….” In this manner, the kindergarten teacher actively translates clinical concepts to a language that seems adjusted to everyday language, as if they were speaking to a parent or the children themselves. Brit also translates, explaining that she has “not have such precise language,” but that psychosocial support means “building good relationships.” This making of alternative definitions and concepts can interestingly be read as an act of communication as if they now are in a dialog with children or non-professionals. As a result, the term psychosocial support is translated, and maybe also diversified. It can give the impression that they are in a dialog with an unseen audience, that does not to be other professionals, but rather parents or children.

### 4.3 Psychosocial support reconceptualized by kindergarten teachers: three main components

During the rest of the interviews, the kindergarten teachers maintain the distance to psychosocial support as a term and do not use it. Instead, one could say that kindergarten teachers *reconceptualize* psychosocial support through three main components of support toward children. These components can be summarized as (a) support of feelings, (b) support of relations and open-ended meetings, (c) support having time.

### 4.4 Component a: support of emotions and feelings

One main component all the kindergarten tea of psychosocial support is the support of emotions and feelings. Children’s feelings seem to be at the core of the work with children in general, and it is one of the most frequent terms used by all kindergarten teachers. Anna says:

” … children are little people full of feelings. Everything they experience in a day, both at home and in kindergarten affects them in some way. They pick up everything”.

The kindergarten teachers do not describe emotions, which often can be described as a psychologist’s terminology (Rogndal, 2017), but the everyday term “feelings”. Anna expresses feelings as a sort of observation of what a child is, also a child’s sensitivity toward people, places, and changing character of feelings during the day. The other teachers describe various professional actions and reactions to children’s feelings.

“All children need psychosocial support in kindergarten, to recognize their feelings, to know about feelings, to be able to assure consideration from others and to others” (Kari).

“[the children] need a lot of validation and caring, from the staff. So they can feel they belong with us, that they feel safe in the kindergarten […]” (Ida)

“There is a need for regulation of feelings, and support in dealing with difficult and strong feelings. If the child is feeling unhappy […] they come to an adult to be comforted, then too […] there are a lot of children who need comforting to control their feelings in both small and large situations in kindergarten” (Anna).

In these quotes, the kindergarten teachers enlist several actions on children’s feelings. They describe knowledge about feelings, as a kind of psycho-educative perspective and use terms like “recognition,” “consideration” and “validation” of feelings. These terms could be elaborated on as philosophical or psychological terms. However, they are only enlisted briefly among all the kindergarten teachers and can indicate a low emphasis on academic terms.

Thus, the kindergarten teachers in this study seem to conceptualize psychosocial support in terms of attention to the children’s feelings. They only mention academic complex terms and seem to prefer to describe intuitively the feelings as what a child is or does. The language is vivid, with everyday affectionate examples, making psychosocial support a matter of sensing, maybe more than registering or systematizing observation.

### 4.5 Component B: support of relational spaces and open-ended meetings

The kindergarten teachers introduce also psychosocial support as a social and relational domain. The term “other” is central for all the kindergarten teachers, in phrases like “each other,” “in relation to others” “other children” “being with/playing with others.” In addition, alternative concepts about the relations, such as “group” and “inclusion among the children,” “interact” and “relationship” make up a major feature throughout the data material. This could imply that kindergarten teachers describe psychosocial support with an emphasis on the social aspect.

Yet, few kindergarten teachers specify for instance the development of social skills, but make summarizing headlines:

“In the form of supporting children’s mental health and interaction with others, self-regulation, taking the other's perspective … coping with feelings, considering the feelings of others, expressing feelings (Kari)

These social skills are presented as a kind of list, and they utilize terms from the psychological discipline, such as emotional regulation and cognitive behavioral approaches (Lekhal and Drugli, 2019) However, only Kari uses these terms, and they are not explained further by Kari or other kindergarten teachers.

The kindergarten teachers do not describe singular children with diagnoses, special needs, or trauma. Instead, all the children in the kindergarten are described in general.

“Psychosocial support … what I think of when I hear this word are children who need support so they can interact with other others, build relationships with others, when the child is struggling, or the situation around the child is difficult […] So the challenge is to include the child in good interactions, good play” (Peter)

According to Peter, the teachers’ job is not to interfere with the emotional difficulties of the child but lies in involving and including the child and securing good play. Being able to relate and the act of inclusion seems to work indirectly as a protective force when a child is struggling.

The social relations and values of belonging and inclusion have a high priority in kindergarten. However, the details of how the kindergarten teacher works with social relations seem not to be emphasized in these interviews. First, this could be interpreted as a lack of vocabulary or process dimensions for facilitating the social relations between the children. Second, relations in terms of belonging and inclusion can adhere to a tacit value more than practical skills. A third way to understand the lack of procedures is, the kindergarten teachers seem to draw attention to the relations in the kindergarten. Psychosocial support is a kind of open-ended meeting and is essential to psychosocial support.

“[…] essentially it’s about meeting the child where they are, on many levels”. (Anna).

“It [psychosocial support] is quite a wide concept, but essentially it’s about meeting the child where they are, on many levels” (Ida)

Strikingly many of the teachers use the word “meet” and “meeting the child where s/he is” in the interviews of this study. The meeting is described in expressions like for instance “being available for the children,” “creating a safe space” and “meeting the children where they are.” This meeting is not framed as a physical meeting. Instead, the meeting appears as something indistinct, like a kind of sensitivity or open-ended presence.

Thus, the relational aspect of psychosocial support does not lie in developing particular social competencies of the child (Lamer, 2014) or in psychological procedures, for instance, emotional regulation of for example how to calm a nervous child (Nordanger and Braarud, 2014). Instead, the relational focus appears with layers of existential and ethical space with a less verbalized notion and open-ended meetings.

### 4.6 Component C: psychosocial support and the quest for time

“Time” or “having time” is emphasized and engages all the kindergarten teachers. In the interviews, time appears not only as an organizational unit of hours and minutes; it can also be read as a kind of open-ended attention for the kindergarten teachers. It might denote a flexibility that seems crucial for providing psychosocial support, both when it comes to practical organization as well as a quality of the relation to the child.

First, the teachers seem to repeatedly use the terms “having time” or “not having time.” Time appears as a face value and practical condition for providing good psychosocial support-or the risk of the opposite. For instance, Ida describes the importance of

“… staff who see how important [psychosocial support] is, even when time is tight. In kindergarten there are many routines, things that need to be done throughout the day. That can feel stressful”.

Time is described as tight, and even something the teachers have or do not have as a crucial factor, adding pressure for providing psychosocial support.

Second, time is also depicted through pedagogical values for the kindergarten teachers. Vera says: “In kindergarten, we say we have “all the time in the world,” but we do not have “all the time in the world”! We always have too few hands”. On the one hand, Vera expresses worries about not having enough time for providing psychosocial support. “Not having time/hands” can be described as a practical and linear understanding of time (Barad, 2017), and can refer to practical and organizational matters in kindergarten. On the other hand, Vera’s statement also exposes another and deeper conflict about time. Saying that “we have all time in the world” implies a professional ideal presented for the children. This value might refer to the understanding of time as relative and flexible in kindergartens, where the time and activities of the child guide the day (Ulla, 2017). Yet, through the limitations of the linear time, Vera reveals foundational professional values under pressure and implicit threats toward the provision of good psychosocial support.

Third, time appears a kind of available to the child, displaying risk and vulnerability in providing psychosocial support, for both the child and the teacher.

“I am very conscious of wanting the child to have the time this child needs to feel ready to explore things again or get back into play. For some children that can take a long time, so not to rush them in, but at the same time think it is good for the child to come in, or that they want to go into play with others. (Peter)

“Some of the children we meet here take a long time to warm to you. Days and weeks can go by where they are just not interested in any form of contact with the teachers. Just grumpy, quarrelsome, won’t join in, nothing is fun, everything is boring, that’s boring, that’s boring, and one just has to keep trying. It means you just must try every day to make contact …” (Brit).

In these quotes, Brit and Peter utter that providing psychosocial support and having contact is not a given, even for them as experienced kindergarten teachers. Peter and Brit both describe patient waiting. Psychosocial support is not only dependent on what they give as teachers but is dependent on waiting for a connection with the child. Brit describes the steady waiting as a strategy for connecting the grumpy and the quarrelsome child. This might take days or weeks or months, revealing time as important for the fragile acts of communication and coexistence for the kindergarten teachers.

Peter says that he waits for “the child to come in” and wants to go into play and join in with the others. Psychosocial support is established as a long-term goal and might lie in the ability to envision a long-term goal of participation and being part of a group.

In the quotes of Peter and Brit above it is not necessarily the teacher who provides psychosocial support. The teacher does not have a clear role in the terms of scripted and defined actions in providing psychosocial support. Instead, they wait: They wait for the child to join, they wait for the child to play, and they wait for the general contact with the teachers. According to this approach, there is a genuine focus on the child as an actor (Hewett, 2001; Bae, 2007; Ministry of Education and Research, 2017). The meeting with the child appears to be an open space for the child to act, connect, and develop. According to such logic, psychosocial support is a matter of promoting a child’s agency and willful actions. Contact and attachment are focused on the acting and the interacting child. From Brit’s quote, the teacher does not correct or regulate the behavior of the grumpy and quarrelsome child. The kindergarten teacher mostly waits. The waiting can represent an open space where children play, quarrel, interact – these seem to be fundamental in the kindergarten teachers’ descriptions of psychosocial support. In the quote, especially Brit displays a vulnerable approach to time. Time does not only imply a practical organizational structure, nor a procedural order or structure of the day. Time rather appears as a relational and open space, a circular matter of repetition and observation (Barad, 2017) whereas anything could happen, and with the risk that both the teacher and the child can fail. Providing psychosocial support is all but a procedure, it turns the attention to agency and subjectivity as the most important assets for conceptualizing psychosocial support.

However, the agency of children’s open space is also threatened.

“Children are pressured – “pressured” into a form dictated by adults and where children have less opportunity to contribute, where children’s participation is marginal, and where the adult's needs are more visible. They [the adults] can take up too much space.” (Anna).

In this quote, Anna displays the vulnerability and the risk in psychosocial support as an open-ended presence., which could be the adult taking up too much space. Maybe she also makes a more general comment about the “adult world” of policies dominating the agenda in kindergarten.

Thus, through the time descriptions, the kindergarten teachers do not only communicate that time is something that organizes the day in terms of the staff-child ratio. Instead, time can represent the possibility of open spaces to enable a child’s agency and subjectivity as the most important assets for conceptualizing psychosocial support. As a summary (Table 2), the kindergarten teacher’s conceptualizations can be summarized by three components and examples of main concepts.

**Table 2:** Summarizing key terms and analytical considerations in the study.

Dimension	Main emphasized concepts, examples	Analytical considerations
Emotional dimension	«Feelings»	The kindergarten teachers emphasize everyday terms and avoid academic terms.
Social dimension	“Meet” and “meeting the child where s/he is,” “each other,” “in relation to others,” “other children” “inclusion among the children”	Psychosocial diagnoses/disorders are not mentioned.
Time–space dimension	“Having time” “having too few hands,” “waiting for the child”	Psychosocial support as a space of risk and vulnerability in the relation to each child.

### 4.7 Professional positioning to psychosocial support: ambiguous professional identities

A striking feature in this study is that the kindergarten teachers seem to avoid the professional title “teacher,” which can be an important part of how the kindergarten teachers position themselves to the notion of psychosocial support. The kindergarten teachers speak about themselves as “the adult,” which is a previously debated issue in Norway (Fimreite and Moxnes, 2021). Not using the professional title appears somehow contradictory to the fact that all the informants hold at least a bachelor’s degree, have additional courses, and are well-experienced teachers with a particular interest in psychosocial support. The kindergarten teachers also express pride in their formal education and confidence in their work through the interviews. The use of the “adult” can rest on more than a pragmatic and communicative title communicating inferiority. Rather, the kindergarten teachers take their pride in the “meeting with the child.” Being “the adult” is then a token for highlighting the focus of the meeting, which is based on silence, patiently waiting for the child, and active promotion of the agency of the child. The agency does not come as a given, especially not in strong asymmetrical relationships (Sorbring and Kuczynski, 2018).

Yet, when it comes to psychosocial support, many of the kindergarten teachers express psychosocial support as if navigating in an unknown field. Even when describing formal education, Anna maintains psychosocial support as a matter of handling *ad hoc* situations. “It wasn’t easy to make a plan and I did not feel I had a lot in my pedagogical bag of knowledge in a way, I felt it a bit *ad hoc*” (Anna). In contrast to the knowledge Anna had from her formal education, the daily work represents challenges, and she expresses that she does not feel prepared. The plans and former pedagogical knowledge are experienced as insufficient, both by Anna and other teachers.

All the teachers in the study also express openness and call for more formal education as well as knowledge of psychosocial support. Peter says, “One is never finished learning!.” However, the sources of knowledge vary. Some of the kindergarten teachers have had updates and other courses on psychological theories. Most of the kindergarten teachers mention a general theory of attachment, like “the circle of security” theory, that has been translated into Norwegian and presented to the kindergarten during the last five years (Brandtzæg et al., 2013). A couple of teachers express the need for more psychological knowledge,

“… to meet children’s need where they are, but also knowledge of children’s brain development, about the three sides of the brain, and where emotions lie, and where experiences are stored, how understanding gets connected to the experience, how late it gets connected, if you look at how children mature” (Anna)

Anna expresses a need for more knowledge and can label headlines of specific fields of developmental psychological knowledge. Yet, at the same time, she maintains that kindergarten teachers “meeting the children where they are” is a basic condition for developing their competence.

Despite the need for knowledge from other instances, kindergarten teachers experience little or weak collaborations outside their kindergarten with the other public services. Vera says: “No, no we do not get much input from outside, we must have confidence in each other, and discuss together […] “. Some of the kindergartens have routines for discussing cases and collaborating with other instances when a child has complex issues. A general tendency is still that the kindergarten teachers describe the collaboration internally in the kindergarten and seem less connected to external knowledge circuits.

Thus, the professional expertise, the kindergarten teachers display an ambiguity when they position themselves in the field of psychosocial support. The teachers do not describe themselves as professionals, but as “adults.” Yet they express pride in their formal education as well as in their experience. They call for collaboration, yet in practice, they are delimited to their internal knowledge circuits and describe how they must confide in their own knowledge and discernment. The kindergarten teachers also call for more knowledge, and very well from interdisciplinary knowledge networks.

## 5 Summary and discussion

This study explores how Norwegian kindergarten teachers conceptualize and negotiate the field of psychosocial support. The present study aims to provide in-depth insights into teachers’ professional logic and conceptualization of psychosocial support, calling for an educational contribution and a “footprint” in interprofessional work in MHPSS interventions.

The qualitative analysis of this study shows that the Norwegian kindergarten teachers seem to neglect and even reject the concept of psychosocial support, while also expressing inferiority and lack of agency toward other professions. Despite high formal competence and in-service training in psychosocial support, the typical psychological and formal terms are avoided prior to everyday language. The kindergarten teachers highlight three main dimensions of psychosocial support; the emotional dimension, the social dimension as well as a “time–space” dimension whereas they describe the immediate needs and relations as vulnerable and at risk. However, the teachers also express the desire for more knowledge, and with proudness they energetically engage in fostering children’s agency and functioning by their everyday descriptions of «meeting with the child».

This study represents a small but strategic sample of qualitative interviews with Norwegian expert teachers in a well-developed and stable structure for providing psychosocial support. The results of this case study show that kindergarten teachers display deep ambiguity toward the concept of psychosocial support. These findings represent both obstacles to and possibilities in the field of MHPSS.

On the one hand, kindergarten teachers in this study tend to reject the concept of psychosocial support as an academic term, and terms like trauma or diagnoses do not capture their attention. The teachers establish psychosocial support with seemingly weak definitions; they dismiss, subvert, and translate typical clinical and academic definitions into descriptive, everyday language. They also express an inferior position to other professions and give the impression of low professional self-consciousness, labeling themselves as “adults” instead of “teachers.” These findings can cause foundational difficulties in a clear framing of any clear “educational footprint.”

However, on the other hand, kindergarten teachers’ responses can also be read as important negotiations and active reconceptualizations of psychosocial support. At the core of psychosocial support is the “meeting with the child.” The meeting with the child is expressed as an open-ended presence of having time, making observations, and providing a finely tuned response to the varied needs of the children throughout the day. Thus, kindergarten teachers seek performative and action-oriented concepts of psychosocial support rather than academic, representative concepts, following a typical pattern of teachers’ conceptualizations (Mulcahy, 2011). Despite their seemingly inferior professional position, kindergarten teachers articulate a clear vision for observing and acting according to the child’s needs and facilitating the child’s agency and functioning. Thus, a summarizing finding from this study of the kindergarten teachers’ conceptualization and negotiation of psychosocial support can be described as everyday performative and finely tuned interactions with the child.

Through this present study, it is possible to envision that kindergarten teachers take a position apart from the established controversies in MHPSS measures in education. The teachers seem to avoid standardized concepts. In their pursuit of the children, their emotions, and needs, they seem to have a strong focus on anything but standardized concepts and measures. Yet, this study shows that kindergarten teachers do not necessarily adopt the criticisms from the academic educational discipline toward the MHPSS field either (e.g.,). Despite rejecting the concept of psychosocial support, they also articulate explicit emotional needs and call for active collaboration.

The findings from this qualitative study indicate several challenges for further development of the educational contribution in the field of MHPSS. The first challenge is directed to MHPSS. The teachers do not necessarily need health-based initiatives as a sole “help to think” (Raknes, 2019), as they do not lack psychological knowledge. Education is not necessarily only a passive “receiver” of health-based interventions. It might also be important to establish systems for including teachers as professional premise providers, as the teachers can provide a systematized, professional body of knowledge and represent comprehensive local knowledge (Ní Chorcora and Swords, 2021).

A first challenge could be directed toward the kindergarten teachers. In a landscape of critical needs for good MHPSS measures, the kindergarten teachers’ approach to psychosocial support might be perceived as too vague, and the kindergarten teachers might seem unknowledgeable. If so, it is easy to understand that more defined and “harder” psychological concepts of, e.g., “emotional regulation,” might easily be incorporated into kindergartens and education. The teachers’ descriptions of the “meeting with the child” can also be read as too dependent on the individual teacher and bound up in time and space, and secrecy and exclusivity. A weak or privatized conceptual basis can challenge coordinated actions for MHPSS, which are also dependent on clear educational perspectives when developing systematic knowledge. It is necessary to dare to develop concepts that move beyond the subjective everyday descriptions, yet without being too standardized and far from educational practices.

A second challenge is directed toward educational research on MHPSS, including ourselves. This study does not only suggest that other sectors should include education as the “weaker part” but challenges also the educational sector for increased participation in conceptualizing the field of MHPSS. This study shows that kindergarten teachers have a clear focus on the psychosocial needs of the child and call for more knowledge of the field of MHPSS. They do not reject MHPSS measures as such but try to find their own approach. Educational research, then, needs to expand rather than hold on to a defensive position toward any other disciplines. Education is not only under attack or “under invasion” toward the field of MHPSS. The pedagogical notions about a child’s agency and experiential learning (Lillejord et al., 2015) are obvious perspectives included well in the field of MHPSS. The challenge for the academic landscape is to develop professional educational concepts that can communicate with an interprofessional audience in the field of MHPSS.

A third challenge is directed toward the health sector in the field of MHPSS. The efforts for MHPSS are depending on increased coordination and inclusive perspectives from education as well as mental health and child welfare services (Nordanger and Braarud, 2014; World Health Organization, 2022a). The success of MHPSS interventions should be evaluated according to how they are rooted in practice-based foundations during their development (Lund et al., 2013; Sashidharan et al., 2016). If cross-sectoral work and good cooperation are the keys to better psychosocial support, it is crucial to develop common professional concepts and active boundary work (Akkerman and Bakker, 2011). There is a need for an “educational footprint,” yet only as one footprint among many.

## Data availability statement

The raw data supporting the conclusions of this article will be made available by the authors, without undue reservation.

## Ethics statement

Ethical approval was not required for the studies involving humans due to non-sensitive data and the level of anomyization. The study was conducted in consultation with SIKT (Norwegian Agency for Shared Services in Education and Research) and accordance with the Norwegian legislation and institutional requirements. The participants provided their written informed consent to participate in this study, and the data material has been sufficiently anonymized according to national regulations. The studies were conducted in accordance with the local legislation and institutional requirements. The participants provided their written informed consent to participate in this study. Written informed consent was obtained from the individual(s) for the publication of any potentially identifiable images or data included in this article.

## Author contributions

IC: Conceptualization, Data curation, Formal analysis, Funding acquisition, Investigation, Methodology, Project administration, Resources, Validation, Writing – original draft, Writing – review & editing.
