# Corrigendum: The call for an “educational footprint” in conceptualizing mental health and psychosocial support: the case of Norwegian kindergarten teachers

**DOI:** 10.3389/fpsyg.2024.1490694

**Published:** 2024-12-10

**Authors:** Ingrid R. Christensen

**Affiliations:** University of South-Eastern Norway (USN), Notodden, Norway

**Keywords:** mental health and psychosocial support, kindergarten, teachers, conceptualization, qualitative study mental health and psychosocial support, qualitative study

In the published article, there was an error. A couple of “X” are remaining from the anonymous version. Instead of “X,” the text should state “Palestine” and “Palestinian,” respectively.

A correction has been made to the **Introduction**, paragraph 8. This sentence previously stated:

“The present study is a development of a previous international comparative study of psychosocial support described by kindergarten teachers in X and Norway. The comparison showed that although X kindergarten teachers were more well-acquainted with the MHPSS terminology, the two countries shared many common challenges in providing psychosocial support in kindergarten.”

The corrected sentence appears below.

“The present study is a development of a previous international comparative study of psychosocial support described by kindergarten teachers in Palestine and Norway. The comparison showed that although Palestinian kindergarten teachers were more well-acquainted with the MHPSS terminology, the two countries shared many common challenges in providing psychosocial support in kindergarten.”

The author apologizes for this error and states that this does not change the scientific conclusions of the article in any way. The original article has been updated.

